# Long non-coding RNA HOTTIP promotes prostate cancer cells proliferation and migration by sponging *miR-216a-5p*

**DOI:** 10.1042/BSR20180566

**Published:** 2018-09-28

**Authors:** Bin Yang, Ge Gao, Zhixin Wang, Daju Sun, Xin Wei, Yanan Ma, Youpeng Ding

**Affiliations:** 1 Department of Breast Surgery, China-Japan Union Hospital of Jilin University, Changchun, Jilin, China; 2 Department of Pathology, China-Japan Union Hospital of Jilin University, Changchun, Jilin, China; 3 Department of Urology, China-Japan Union Hospital of Jilin University, Changchun, Jilin, China

**Keywords:** ceRNA, EMT, HOTTIP, LncRNA, miR-216a-5p, PCa

## Abstract

Long non-coding RNAs (lncRNAs) are a class of ncRNAs with >200 nts in length that regulate gene expression. The HOXA transcript at the distal tip (HOTTIP) lncRNA plays an important role in carcinogenesis, however, the underlying role of HOTTIP in prostate cancer (PCa) remains unknown. The aim of the present study was to evaluate the expression and function of HOTTIP in PCa. In the present study, we analyzed HOTTIP expression levels of 86 PCa patients in tumor and adjacent normal tissue by real-time quantitative PCR (qPCR). Knockdown or overexpression of HOTTIP was performed to explore its roles in cell proliferation, migration, invasion, and cell cycle. Furthermore, bioinformatics online programs predicted and luciferase reporter assay were used to validate the association of HOTTIP and *miR-216a-5p* in PCa cells. Our results found that HOTTIP was up-regulated in human primary PCa tissues with lymph node metastasis. Knockdown of HOTTIP inhibited PCa cell proliferation, migration, and invasion. Overexpression of HOTTIP promoted cell proliferation, migration, and invasion of PCa cells. Bioinformatics online programs predicted that HOTTIP sponge *miR-216a-5p* at 3′-UTR with complementary binding sites, which was validated using luciferase reporter assay. HOTTIP could negatively regulate the expression of *miR-216a-5p* in PCa cells. Above all, the knockdown of HOTTIP could represent a rational therapeutic strategy for PCa.

## Introduction

Prostate cancer (PCa) is a common malignancy in which affected individuals are at risk of death by cancer-related causes [1]. Current treatments include radical prostatectomy, radiotherapy, and chemotherapy. There is currently no effective systemic or topical treatment for metastatic castration-resistant PC (mCRPC) [2,3]. The emergence of molecularly focussed approaches to cancer has fundamentally changed the path to diagnosis and treatment of malignancies. Therefore, it is urgent to understand the molecular mechanisms of PCa, and to develop the effective therapeutic approaches.

Long non-coding RNAs (lncRNAs) are a class of ncRNAs with >200 nts in length that regulate gene expression [4]. In humans, lncRNAs have emerged in the regulation of progression of several tumor types [5]. LncRNAs could act as competing endogenous RNAs (ceRNAs) with miRNAs to play a post-transcriptional regulatory role in the gene expression [6]. The lncRNA HOXA transcript at the distal tip (HOTTIP) located at the 5′-end of the HOXA cluster, which is a key locus control element of *HOXA* genes, and is brought into close proximity to the 5′ HOXA genes by chromosomal looping [7]. HOTTIP has been suggested as a potential prognostic biomarker for diverse cancers [8–12]. However, HOTTIP aberrantly expressed in PCa cells and involved in controlling PCa progression remains largely unknown. In the present study, we investigated the functions of HOTTIP in PCa, especially to explore its role in proliferation and metastasis.

## Materials and methods

### Computational analysis

Two human lncRNA microarray datasets (GSE73397, https://www.ncbi.nlm.nih.gov/geo/query/acc.cgi?acc=GSE73397 and GSE55909, https://www.ncbi.nlm.nih.gov/geo/query/acc.cgi?acc=GSE55909) were obtained from public database, NCBI [13,14]. Aberrantly expressed lncRNAs were identified using Venn analysis and co-expression network analysis [15].

### Patient samples

PCa tissues and paired adjacent non-cancerous tissues were obtained from surgical resection at China-Japan Union Hospital of Jilin University. Both tumors and non-cancerous tissues were immersed immediately in RNA solution later overnight, and then stored at −80°C. Prior to the use of these clinical materials for research purposes, written consent from all patients and approval of China-Japan Union Hospital of Jilin University Ethic Review Committees were obtained.

### Cell lines

PCa cell lines DU145, PC3, LNCaP, and 22Rv1 were purchased from American Type Culture Collection (ATCC, Rockville, MD). PCa cell lines were cultured in RPMI-1640 or minimum essential Eagle’s medium, supplemented with 10% FBS and antibiotics. The human non-tumorigenic prostate epithelial cell line RWPE1 was cultured in keratinocyte serum-free medium supplemented with 5 ng/ml human recombinant epidermal growth factor and 30 mg/ml bovine pituitary extract (Invitrogen, Carlsbad, CA). Cell lines were maintained in a 5% CO_2_ humidified atmosphere at 37°C.

### RNA isolation and quantitative real-time PCR

Quantitative real-time reverse transcription (qRT)-PCR (qRT-PCR) was used to detect the expression of HOTTIP in tumor tissues and cell lines. Total RNA was extracted using the TRIzol reagent (Invitrogen). RNA was reverse transcribed into cDNA using a Reverse Transcription Kit (Takara, Dalian, P.R. China). Then cDNA was used as a template with the GoTaq (quantitative PCR) qPCR Master Mix kit (Promega, Madison, WI, U.S.A.) and the reaction was monitored in an Mx3005P QPCR System (Stratagene, La Jolla, CA, U.S.A.). In the present study, the housekeeping gene glyceraldehyde*-*3-phosphate dehydrogenase (*GAPDH*) was used as a reference to normalize the threshold cycle (*C*q) value. Primers were synthesized by Sangon Biotech (Shanghai, P.R. China) and their sequences were as follows: HOTTIP: sense: 5′-GTGGGGCCCAGACCCGC-3′; antisense: 5′-AATGATAGGGACACATCGGGGAACT-3′, and GAPDH: sense: 5′-ACCCACTCCTCCACCTTTGAC-3′; antisense: 5′-TGTTGCTGTAGCCAAATTCGTT-3′.

### Transfection

To knockdown the HOTTIP expression, the siRNA and non-specific control siRNA were synthesized (Carlsbad, California, U.S.A.) and transfected into cells using Lipofectamine 2000 (Invitrogen, U.S.A.). To overexpress HOTTIP, the full length coding sequence for HOTTIP was amplified and subcloned into the pcDNA 3.1(+) vector (Invitrogen) according to the manufacturer’s instructions. Cells were transfected with a negative control vector or the HOTTIP-expressing plasmid according to the manufacturer’s protocol. Cells were harvested after 48 h for qRT-PCR analyses.

### Cell proliferation assay

Cell viability was measured by Cell Counting Kit-8 assay (CCK8, Dojindo Molecular Technologies). Briefly, cells were plated at a density of 5 × 10^3^ cells/well on 96-well plates and subjected to different treatments. Following 48-h incubation at 37°C in a humidified atmosphere containing 5% CO_2_, cells were incubated for another 2 h with CCK8 reagent. The absorbance was determined at 450 nm using FLx800 Fluorescence Microplate Reader (Biotek).

### Cell cycle analysis

The cells were washed in PBS and fixed in 75% ice-cold ethanol at −20°C overnight. After rehydration with ice-cold PBS, cells were stained with PI/RNase Staining Buffer (BD Biosciences, San Jose, CA, U.S.A.) and analyzed using flow cytometry on a FACSCalibur Flow Cytometer (BD Biosciences) using the CellQuest Pro software (BD Biosciences).

### Matrigel invasion assay

Cells were seeded into 24-well Matrigel Invasion Chambers (Corning, NY, U.S.A.) with polyethylene terephthalate membrane containing 8.0-μm pores. As a chemotaxis factor, 10% FBS was added to the lower compartment of the chambers. After incubation for 24 h at 37°C, all cells that did not enter the filter were removed gently by scraping the upper side of the filter with a wet cotton swab. Cells that migrated to the bottom filter surface were fixed by soaking the insert in MeOH for 3 min, stained with Hematoxylin and Eosin, and air dried. Filters cut from inserts were mounted upside down on glass slides. Invading cells were counted under a light microscope.

### Western blotting assay

Total proteins were extracted from PCa cells using RIPA lysis and extraction buffer (Thermo Fisher Scientific). Protein concentrations were measured using a BCA Protein Assay Kit (Thermo Fisher Scientific). Standard Western blotting techniques, and the Bio-Rad ChemiDoc MP imaging system (Hercules, CA, U.S.A.) were used. The primary antibodies included rabbit anti E-cadherin, anti-N-cadherin, anti-Vimentin (Santa Cruz Biotechnology, Santa Cruz, CA, U.S.A.), and antihuman GAPDH (CST). The secondary antibody was goat anti-rabbit IgG (Invitrogen). Protein levels were measured by gray value with Quantity One software.

### Luciferase assay

A dual luciferase assay kit (Beyotime Biotechnology) was used for dual luciferase assay. Briefly, cells were co-transfected with *miR-216a-5p* or *miR-control* and pMIR-report luciferase vector containing 3′ UTR of POU2F1, wild-type or mutant HOTTIP fragment, using Lipofectamine 2000 (Invitrogen). Cells were collected and lyzed for luciferase detection 48 h after transfection. The relative luciferase activity was normalized against the *Renilla* luciferase activity.

### Statistical analysis

Continuous variables were expressed as means ± S.D.; differences were assessed for significance using Student’s *t* test or the Mann–Whitney test. Categorical variables were evaluated using chi-square or Fisher’s exact tests, as appropriate. A *P*-value <0.05 was considered statistically significant. All tests were performed on SPSS Statistics 20.0 software.

## Results

### HOTTIP was up-regulated in human PCa tissues and cell lines

**Figure 1:**
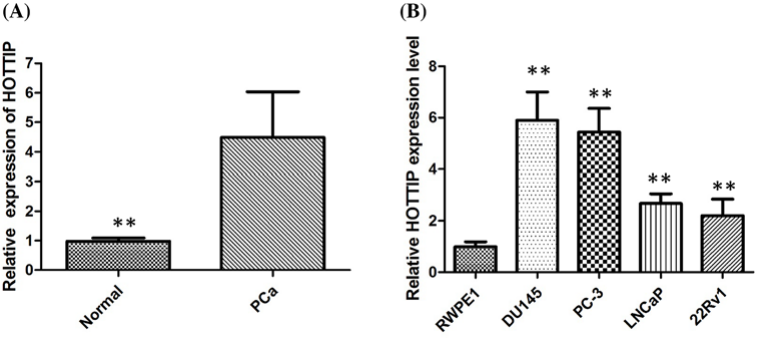
HOTTIP was up-regulated in human PCa tissues and cell lines (**A**) HOTTIP was detected in PCa tissues and adjacent non-cancerous tissues by qRT-PCR; (**B**) qRT-PCR showing expression level of HOTTIP in PCa cell lines. **P < 0.01.

To identify the levels of HOTTIP in PCa, we used the qPCR assay to analyze the expression of HOTTIP in 86 pairs of PCa tumor tissues. We first found that HOTTIP was up-regulated in PCa tissues when compared with normal tissues (*P*<0.001, Figure 1A). Furthermore, HOTTIP expression was significantly associated with several clinical pathological features such as TNM stage (*P*<0.001) and tumor size (*P*=0.016), but not with age. We further used qRT-PCR to demonstrate the expression of HOTTIP in a panel of human PCa cell lines, and we found that its levels in four human PCa cell lines DU145, PC3, LNCaP, and 22Rv1, were higher than the levels in the RWPE1 human normal prostate epithelial cell line (*P*<0.01, Figure 1B). We found that DU145 and PC3 cells had higher expression of HOTTIP, but 22RV1 cells had lower expression of HOTTIP. Thus, we used these three cells as a surrogate model system to investigate the biological role HOTTIP in PCa cells.

### Influence of HOTTIP on cellular proliferation and cell cycle

**Figure 2:**
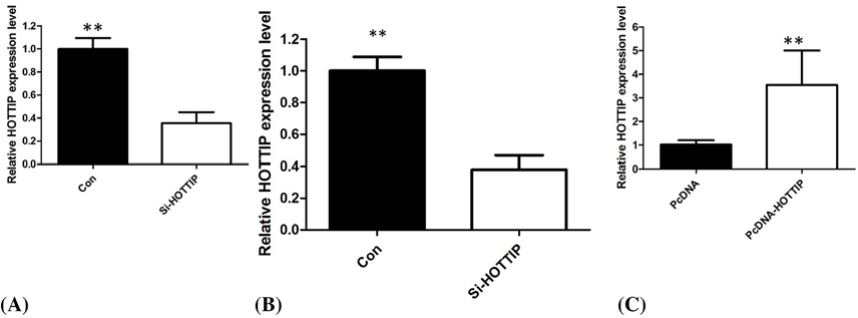
siRNA was employed to knockdown HOTTIP and expressing plasmid to overexpress HOTTIP in PCa cell lines siRNA was employed to knockdown HOTTIP expression in DU145 cells (**A**) and PC3 cells (**B**). Expression plasmid was employed to upregulate HOTTIP expression in 22RV1 cells (**C**). **P < 0.01.

**Figure 3:**
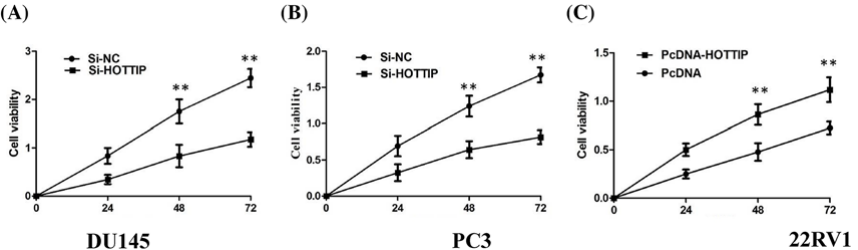
Influence of HOTTIP on cellular proliferation (**A**) CCK8 assay showing knockdown of HOTTIP inhibited cell proliferation of DU145 cells; (**B**) CCK8 assay showing knockdown of HOTTIP inhibited cell proliferation of PC3 cells; (**C**) CCK8 assay showing overexpression of HOTTIP promoted cell proliferation of 22RV1 cells.**P < 0.01.

**Figure 4:**
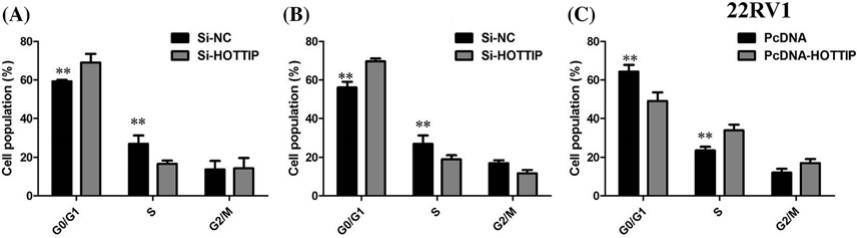
Influence of HOTTIP on cell cycle (**A**) DU145 cells transfected with si-HOTTIP all had cell-cycle arrest at the G_1_/G_0_ phase compared with cells transfected with si-NC; (**B**) PC3 cells transfected with si-HOTTIP had cell-cycle arrest at the G_1_/G_0_ phase compared with cells transfected with si-NC. (**C**) Enhanced HOTTIP expression increased the S-phase percentage and decreased G_0_/G_1_ phase percentage of 22RV1 cellsAbbreviation: si-NC, siRNA negative control**P < 0.01.

We performed functional analyses to further investigate the effects of HOTTIP on cellular proliferation and cell cycle of PCa *in vitro*. We employed siRNA to knockdown the HOTTIP expression in DU145 and PC3 cells, and up-regulated HOTTIP expression using the expression plasmid in 22RV1 cells (*P*<0.01, Figure 2A–C). The results of CCK8 assay demonstrated that HOTTIP knockdown cells compared with control cells have lower cell proliferation rates in DU145 and PC3 cells (*P*<0.01, Figure 3A,B), enhanced HOTTIP expression in 22RV1 cells showed increased ability of proliferation of 22RV1 cells (*P*<0.01, Figure 3C). Because HOTTIP knockdown inhibited cell proliferation, we investigated whether HOTTIP affects the cell cycle. The results from flow cytometry showed that the suppression of HOTTIP in DU145 and PC3 cells modulated the cell cycle by inducing G_0_/G_1_ arrest when compared with control group (*P*<0.01, Figure 4A,B). Enhanced HOTTIP expression increased the S-phase percentage and decreased G_0_/G_1_ phase percentage of 22RV1 cells (*P*<0.01, Figure 4C).

### HOTTIP regulates invasion and EMT of PCa *in vitro*

**Figure 5:**
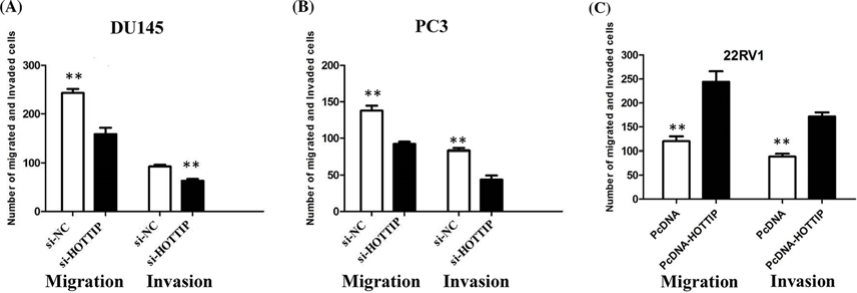
HOTTIP regulates cell migration and invasion of PCa (**A**) Inhibition of migration and invasion of DU145 cells by HOTTIP siRNA; (**B**) inhibition of migration and invasion of PC3 cells by HOTTIP siRNA; (**C**) overexpression of HOTTIP promoted migration and invasion of 22RV1 cells.**P < 0.01.

**Figure 6:**
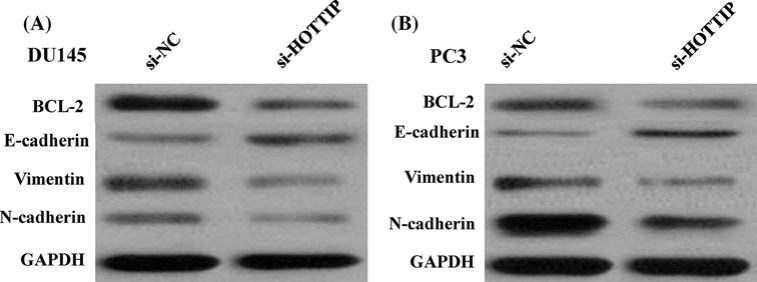
HOTTIP regulates EMT of PCa in vitro (**A**) Knockdown of HOTTIP reverses EMT in DU145 cells; (**B**) knockdown of HOTTIP reverses EMT in PC3 cells.

As cell invasiveness ability is one of the hallmarks of cancer metastasis, we asked whether the overexpression of HOTTIP would display a more invasive phenotype as assessed by a matrigel invasion assay. In DU145 and PC3 cells, the number of cells transferred from the upper chamber to the lower chamber decreased in the HOTTIP knockdown groups compared with the NC groups (*P*<0.05; Figure 5A,B). Conversely, HOTTIP-overexpressing 22RV1 cells presented with increased number of cells transferred from the upper chamber to the lower chamber (*P*<0.05; Figure 5C). Western blot analysis with anti-Vimentin, anti N-cadherin, and anti-E-cadherin antibodies demonstrates that cell line DU145 and PC3 of HOTTIP knockdown have diminished Vimentin and N-cadherin protein expression and enhanced E-cadherin expression compared with the control cell lines (Figure 6A,B).

### HOTTIP may act as a ceRNA of *miR-216a-5p* in PCa cell

**Figure 7:**
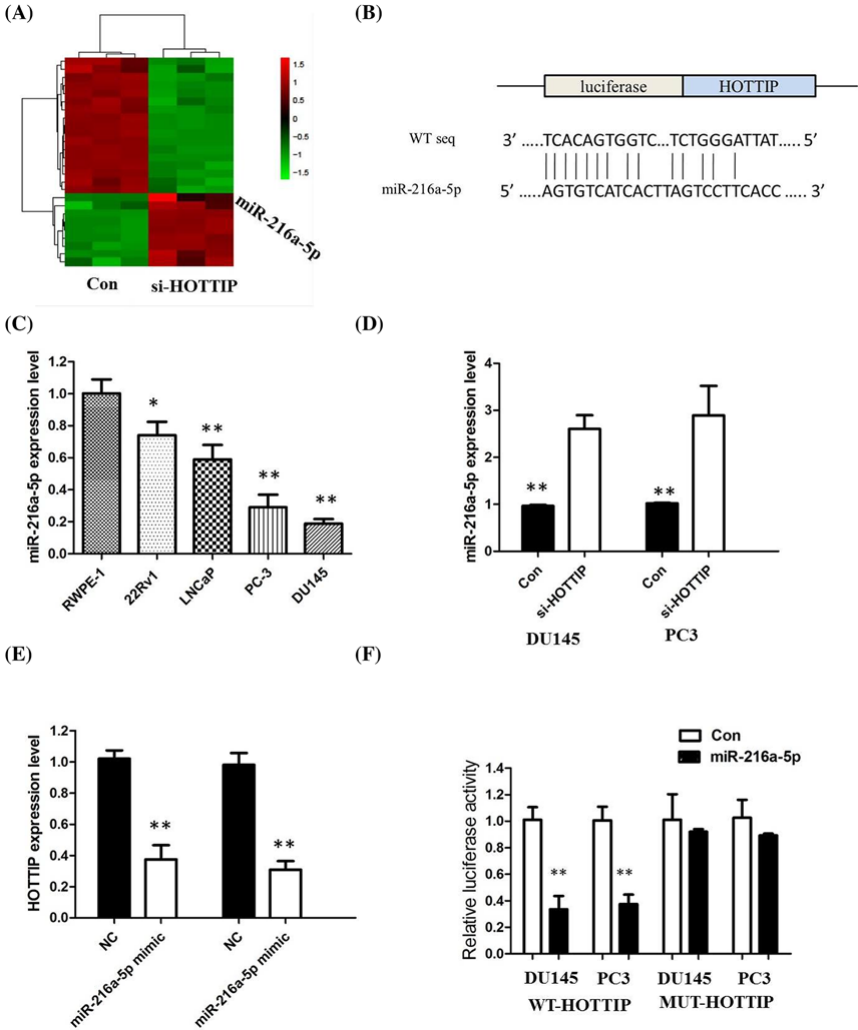
HOTTIP may act as a ceRNA of miR-216a-5p in PCa cell (**A**) DU145 cells were transfected with si-HOTTIP for 48 h. Hierarchical clustering revealed systematic variations in the expression of *miRNAs*. Numerous differentially expressed miRNAs between control and si-HOTTIP transfected DU145 cells are shown on a scale from green (low) to red (high). (**B**) StarBase v2.0 results showing the sequence of HOTTIP with highly conserved putative *miR-216a-5p*-binding sites. (**C**) Expression levels of *miR-216a-5p* in different PCa cell lines were determined by qRT-PCR. (**D**) Silencing of HOTTIP increased the expression level of *miR-216a-5p* in DU145 and PC3 cells. (**E**) *miR-216a-5p* inhibited the expression of HOTTIP in DU145 and PC3 cells. (**F**) The wild-type or mutant *miR-216a-5p*-binding sites in HOTTIP were inserted into pMIR-report luciferase vector. Luciferase activity was detected in PCa cells co-transfected with *miR-216a-5p* or negative control (miR-control) and reporter plasmids containing WT-HOTTIP (wild-type) or MUT-HOTTIP (mutant type). The normalized luciferase activity in the miR-control group was used as the relative luciferase activity. All tests were at least performed three times. Data were expressed as mean ± S.D. *P<0.05, **P<0.01.

To investigate the effect of HOTTIP on the expression of miRNA*s*, DU145 cells were transfected with si-HOTTIP for 48 h. As shown in Figure 7A, miRNA microarray was performed after HOTTIP knockdown, and *miR-216a-5p* was screened out by setting threshold values (FC < −2, *P*<0.01, *n*=3). Bioinformatics prediction with TargetScan and miRDB of miRNA recognition sequences on HOTTIP revealed the presence of *miR-216a-5p*-binding sites, and the potential binding sites were shown in Figure 7B. Next, we measured the levels of *miR-216a-5p* expression in various PCa cell lines. As shown in Figure 7C, the expression of *miR-216a-5p* was obviously decreased in DU145 and PC3 cells, indicating the opposite result to HOTTIP expression.

To explore the regulatory relationship between HOTTIP and *miR-216a-5p*, DU145 and PC3 cells were transfected with either empty vector or si-HOTTIP, and qRT-PCR was used to detect the expression level of *miR-216a-5p*. The results showed that silencing of HOTTIP increased the expression level of *miR-216a-5p* in DU145 and PC3 cells (*P*<0.01, Figure 7D). Meanwhile, *miR-216a-5p* also inhibited the expression of HOTTIP in DU145 and PC3 cells (*P*<0.01, Figure 7E). Sun et al. [14] found that the anti-apoptotic gene *BCL-2* was the target gene of *miR-216a*. In the present study, we also verified the regulation relationship by Western blot in DU145 and PC3 and found that knockdown HOTTIP expression significantly decreased BCL-2 at protein level (Figure 6A,B).

For further confirmation, we used the luciferase assay to detect the association between HOTTIP and *miR-216a-5p*. We cloned the wild-type sequence of HOTTIP or its mutant sequence into the pMIR luciferase reporter, and co-transfected into DU145 and PC3 cells, the reporter plasmid (or the corresponding mutant reporter) and *miR-216a-5p*. The results showed that the relative luciferase activity of the pMIR-HOTTIP-wt construct was significantly decreased but was abolished in HOTTIP-mt-transfected cells (*P*<0.01, Figure 7F). All these data demonstrated that HOTTIP associated with the *miR-216a-5p* and may function as a ceRNA in PCa cells.

## Discussion

LncRNAs have been shown to aberrantly express in various cancers and involved in carcinogenesis [15–19]. However, to our knowledge, the role of lncRNAs in PCa remains poorly understood and is limited to a few samples that were analyzed [20–23]. Increased HOTTIP expression has been reported in various types of human cancers [23,24]. HOTTIP acted as a potential oncogene which could enhance cell proliferation, migration, and reduce apoptosis [25]. However, the functions of HOTTIP in PCa were previously unknown. In the present study, we demonstrated that HOTTIP is up-regulated in PCa tissues compared with normal controls as well as in PCa cell lines, i.e. DU145 and PC3 cells. Furthermore, inhibition of HOTTIP inhibited cell proliferation, arrested cells at the G_0_/G_1_ phase, and suppressed cell migration. Our study provided a potential therapeutic target for the treatment of PCa.

HOTTIP in PCa patients was associated with an increased tumor size and advanced TNM stage. Previous study reported that knockdown of HOTTIP attenuated hepatocellular carcinoma cell proliferation *in vitro* and markedly abrogated tumorigenicity *in vivo*. To further understand the mechanism of HOTTIP in PCa progression, *in vitro* experiments were conducted. We found that all knockdown HOTTIP PCa cell lines displayed a significant proliferation defect comparedwith negative control cells. Accordingly, flow cytometry analysis indicated that HOTTIP depletion in both cancer cell lines increased the proportion of cells that underwent proliferation arrest.

In addition to investigating whether HOTTIP is implicated in migratory ability under inhibition of HOTTIP, we found that the knockdown of HOTTIP affects migratory ability of DU145 and PC3 cells via EMT activity. HOTTIP knockdown decreased the expression of Vimentin and Snai1 and increased E-cadherin expression. These findings revealed that HOTTIP might be involved in PCa progression and contributed to molecular targetted therapy.

LncRNAs are potentially implicated in determining the cancer development through binding with miRNAs that have a wide range of targets. Zhang et al. [26] found that HOTTIP plays essential role in hypoxia-induced EMT and metastasis of glioma by regulating the *miR-101/ZEB1* axis. To investigate whether HOTTIP can play a role as ceRNA in PCa, we performed a microarray to screen the HOTTIP-regulated miRNAs in PCa searched for the potential interactions with miRNAs by bioinformatic analysis. By luciferase reporter assay, we confirmed that *miR-216a-5p* could directly bind to HOTTIP, and co-transfection luciferase reporter assay revealed that HOTTIP could reverse the inhibited reporter plasmid luciferase activity, indicating that HOTTIP can act as endogenous ‘sponge’ for *miR-216a* in PCa cells.

In conclusion, our findings indicated that the expression level of HOTTIP has the potential to be an oncogene for PCa. Our results provided new insights into the function of lncRNAs in the development of PCa and suggested that HOTTIP represents a potential therapeutic target and prognostic biomarker for PCa.

## Abbreviations

CCK8: cell counting kit-8
ceRNA: competing endogenous RNA
EMT: epithelial-mesenchymal transition
GAPDH: glyceraldehyde*-*3-phosphate dehydrogenase
HOTTIP: HOXA transcript at the distal tip
lncRNA: long non-coding RNA
nc: negative control
ncRNA: non-coding RNA
PCa: prostate cancer
qPCR: quantitative PCR
qRT-PCR: quantitative real-time reverse transcription-PCR
